# Steady-state $\dot{V}{\text{O}}_{2}$ above MLSS: evidence that critical speed better represents maximal metabolic steady state in well-trained runners

**DOI:** 10.1007/s00421-021-04780-8

**Published:** 2021-08-05

**Authors:** Rebekah J. Nixon, Sascha H. Kranen, Anni Vanhatalo, Andrew M. Jones

**Affiliations:** Sport and Health Sciences, University of Exeter, St. Luke’s Campus, Heavitree Road, Exeter, EX12LU UK

**Keywords:** Endurance, Physiology, Oxygen uptake, Performance, Lactate, Threshold

## Abstract

The metabolic boundary separating the heavy-intensity and severe-intensity exercise domains is of scientific and practical interest but there is controversy concerning whether the maximal lactate steady state (MLSS) or critical power (synonymous with critical speed, CS) better represents this boundary. We measured the running speeds at MLSS and CS and investigated their ability to discriminate speeds at which $\dot{V}{\text{O}}_{2}$ was stable over time from speeds at which a steady-state $\dot{V}{\text{O}}_{2}$ could not be established. Ten well-trained male distance runners completed 9–12 constant-speed treadmill tests, including 3–5 runs of up to 30-min duration for the assessment of MLSS and at least 4 runs performed to the limit of tolerance for assessment of CS. The running speeds at CS and MLSS were significantly different (16.4 ± 1.3 vs. 15.2 ± 0.9 km/h, respectively; *P* < 0.001). Blood lactate concentration was higher and increased with time at a speed 0.5 km/h higher than MLSS compared to MLSS (*P* < 0.01); however, pulmonary $\dot{V}{\text{O}}_{2}$ did not change significantly between 10 and 30 min at either MLSS or MLSS + 0.5 km/h. In contrast, $\dot{V}{\text{O}}_{2}$ increased significantly over time and reached $\dot{V}{\text{O}}_{2\,\,\max }$ at end-exercise at a speed ~ 0.4 km/h above CS (*P* < 0.05) but remained stable at a speed ~ 0.5 km/h below CS. The stability of $\dot{V}{\text{O}}_{2}$ at a speed exceeding MLSS suggests that MLSS underestimates the maximal metabolic steady state. These results indicate that CS more closely represents the maximal metabolic steady state when the latter is appropriately defined according to the ability to stabilise pulmonary $\dot{V}{\text{O}}_{2}$.

## Introduction

In a pioneering study, Whipp and Wasserman (1972) reported that, for a given individual, exercise at different constant work rates evoked distinctive response profiles for pulmonary O_2_ uptake ($\dot{V}{\text{O}}_{2}$). It is now recognised that, following the initial cardiopulmonary and fundamental phases, $\dot{V}{\text{O}}_{2}$ might: (1) reach a rapid (i.e. within ~ 3 min of the onset of exercise) steady state; (2) reach a delayed (within ~ 10–20 min) steady state; or (3) not attain a steady state at all, but rather rise with time until the $\dot{V}{\text{O}}_{2\,\,\max }$ is attained, with task failure occurring shortly thereafter (Jones and Poole 2005; Whipp and Ward 1992). These characteristic $\dot{V}{\text{O}}_{2}$ response profiles are emblematic of exercise intensity domains which have been termed moderate, heavy and severe (Carter et al. 2002; Poole et al. 1988; Pringle et al. 2003; Wilkerson et al. 2004). These three exercise intensity domains have been considered to be partitioned by the lactate threshold (LT) or gas exchange threshold for the moderate-to-heavy intensity boundary, and the critical power (CP) or maximal lactate steady state (MLSS) for the heavy-to-severe intensity boundary (Jones et al. 2010; Whipp and Wasserman 1972).

It has been established that the neuromuscular, metabolic, blood acid–base and pulmonary gas exchange responses differ in the three intensity domains, resulting in differences in the predominant causes of fatigue and the corresponding limitations to exercise performance (Black et al. 2017; Burnley et al. 2012; Vanhatalo et al. 2016). The heavy-to-severe intensity boundary is particularly important for endurance exercise in that it will determine whether a particular power output or speed is, or is not, sustainable in a metabolic steady state. Accurately discriminating the boundary between the heavy-intensity and severe-intensity exercise domains is, therefore, of both scientific and practical interest (Burnley and Jones 2018; Jones and Vanhatalo 2017; Vanhatalo et al. 2011).

As noted above, there are two principal approaches for the determination of the heavy-to-severe exercise intensity boundary. The first of these is based upon the well-known hyperbolic relationship between power (or speed) and time, with the asymptote of this relationship representing CP (or critical speed, CS) and the curvature constant (W´ or D´, for cycling and running, respectively) representing a finite amount of work that can be done, or distance that can be covered, above the CP or CS, respectively (Hughson et al. 1984; Monod and Scherrer 1965; Moritani et al. 1981; Poole et al. 1988). Exercise performed above CP results in the development of a pronounced $\dot{V}{\text{O}}_{2}$ ‘slow component’ that does not level off but which eventually drives $\dot{V}{\text{O}}_{2}$ to its maximum value; simultaneously, the intramuscular concentration of phosphocreatine (PCr) falls and the intramuscular concentrations of inorganic phosphate and H^+^ rise inexorably, to reach values that are presumably limiting to muscle function at the limit of tolerance (Black et al. 2017; Jones et al. 2008; Vanhatalo et al. 2016). Muscle and blood lactate concentrations are elevated and also display non-steady-state behaviour above CP, but are elevated (compared to resting values) but stable over time in the heavy-intensity domain below CP (Black et al. 2017; Vanhatalo et al. 2016). The second approach to determine the heavy-to-severe intensity boundary is based solely upon the blood lactate responses to continuous exercise and the identification of MLSS, with the latter being defined as the highest power or speed that can be sustained without a greater than 1 mM increase in blood lactate concentration between 10 and 30 min of exercise (Beneke and von Duvillard 1996; Dekerle et al. 2003; Pringle and Jones 2002; Smith and Jones 2001).

CP and MLSS have often been considered to represent the same phenomenon and the terms are frequently used interchangeably. However, small but statistically significant differences between CP and MLSS, with CP being higher, are frequently reported (Dekerle et al. 2003, 2005; Mattioni Maturana et al. 2016; Pringle and Jones 2002; Smith and Jones 2001) and this has led to debate and over which of them should be considered the ‘gold standard’ (Jones et al. 2019a). The rationale underpinning the specific definition of MLSS (i.e. less than a 1 mM increment in blood [lactate] between 10 and 30 min) is obscure and the protocol from which MLSS is derived, it has been argued, is methodologically biased towards an underestimation of the ‘true’ maximal metabolic steady state (Jones et al. 2019a). Moreover, it has been proposed that using changes in blood [lactate] alone as a proxy index of muscle metabolic and respiratory homeostasis lacks precision and that the profile of pulmonary $\dot{V}{\text{O}}_{2}$ represents a more reasonable ‘global’ index of physiological conditions which are steady state or non-steady state (Jones et al. 2019a). Nevertheless, it is feasible that modification of the definition of MLSS via different permutations of permissible blood [lactate] increment and the time over which lactate accumulates might result in closer agreement between MLSS and CP and enable better approximation of CP from measurements of blood [lactate] derived from submaximal exercise tests.

The purpose of the present study was to investigate, in well-trained runners, which of CS and MLSS better represent the highest speed at which $\dot{V}{\text{O}}_{2}$ can be stabilised (i.e. maximal metabolic steady state). We hypothesised that: (1) CS would be significantly higher than MLSS; (2) CS would more closely approximate the maximal metabolic steady state, as defined via the behaviour of $\dot{V}{\text{O}}_{2}$; and (3) modifying the definition of MLSS would eliminate the difference between CS and MLSS.

## Methods

### Participants

Ten well-trained, competitive male athletes (runners *n* = 7, triathletes *n* = 3; mean ± SD age 22.8 ± 4.8 years, height 1.80 ± 0.05 m, body mass 73.7 ± 5.8 kg, $\dot{V}{\text{O}}_{{2\,\,{\text{peak}}}}$ 63.0 ± 4.0 mL/kg/min) volunteered to participate in this study. The participants gave written informed consent and completed and signed a PAR-Q form as a declaration of their eligibility to take part in the study. Participants had no known medical conditions that would inhibit their ability to perform strenuous exercise or cause them harm while doing so. The study adhered to the principles of the Declaration of Helsinki (2013) and was approved by the University of Exeter Sport and Health Sciences Ethics Committee.

### Experimental design and general procedures

This experiment was designed to investigate the relationship between running speeds at MLSS and CS and to explore which of MLSS and CS provided the better approximation of the maximal metabolic steady state, as defined by the ability to separate running speeds at which $\dot{V}{\text{O}}_{2}$ can, or cannot, be stabilised. To this end, following an initial step incremental treadmill test, participants completed a series of constant-speed treadmill runs of up to 30-min duration or to the limit of tolerance for the assessment of MLSS (3–5 runs) and CS (4–6 runs). Tests for determination of MLSS and CS were numbered consecutively from 1. The order of tests was randomised for each participant in a counterbalanced manner, with five participants starting with a CS test and the other five starting with a MLSS test. The final lab visit involved participants running just above their CS to the limit of tolerance.

The study had a single blind, counterbalanced and randomised design and included 9–12 lab visits. Participants were instructed to avoid strenuous exercise for 24 h before, and caffeine and alcohol consumption for 12 h before, lab visits. Testing was completed at the same time of day (± 2 h) for each participant to minimise any influence of circadian rhythm. All participants were offered a familiarisation visit. Testing was completed within 3–6 weeks, with 2–4 tests per week and at least 24 h between tests. The treadmill (Woodway PPS 55 Sport, Woodway GmbH, Weil am Rhein, Germany) was set to 1% incline for all tests (Jones and Doust 1996). Pulmonary gas exchange was measured during all tests, with participants wearing a facemask connected to a calibrated Jaeger Oxycon Pro breath-by-breath ergospirometry system (VIASYS Healthcare GmbH, Hoechberg, Germany). Heart rate (HR) was also recorded during all tests (Polar T31 Heart rate strap, Finland). Blood [lactate] was measured during the incremental test and the MLSS determination tests. Blood was collected from a fingertip using a single use lancet. The first drop of blood was wiped using a clean tissue then the blood sample was collected in a capillary tube and analysed enzymatically (YSI 2500 Lactate Analyser, YSI, Letchworth, UK).

Before all testing sessions, participants completed a standardised warm up which involved running for 5 min at 10 km/h and the opportunity to stretch. For CS prediction tests > 18 km/h, participants also completed a 30–60 s ‘strider’ at a speed above 10 km/h but below their LT. Time was recorded for all tests to the nearest second. Timing (Traceable Timer, Fisher Scientific, Loughborough, UK) started as participants let go of the treadmill handrails to transition to the moving belt and stopped when they stepped to the sides of the treadmill to end the test.

### Initial incremental test

On their first visit to the laboratory, the participants completed a multi-stage incremental step test to the limit of tolerance. The test commenced with 3 min of standing rest during which pulmonary gas exchange, HR and blood [lactate] were measured. The treadmill test then commenced at a speed of 10–12 km/h, depending on the ability of the participant. Every 3 min, the participant stopped running by stepping to the side of the treadmill belt for 30 s to facilitate the collection of a fingertip blood sample. Speed was increased by 1 km/h every 3 min until the participant could not complete a stage or declined the opportunity to start a new stage. The final completed stage was defined as the peak speed attained. $\dot{V}{\text{O}}_{{2\,\,{\text{peak}}}}$ was defined as the highest 30 s mean value recorded during the test. The running speeds at LT (i.e. the first increase in blood [lactate] above baseline values of ~ 1 mM) and lactate turnpoint (LTP; i.e. a sudden and sustained second threshold increase in blood [lactate] at ~ 2.5–4 mM) were determined visually from plots of blood [lactate] against running speed by two experienced researchers (Jones 2007; Jones et al. 2021).

### Determination of running speed at maximal lactate steady state

Participants ran at a constant (but different) speed for up to 30 min on 3–5 occasions (3 runs for four participants, 4 runs for five participants and 5 runs for one participant). Every 5 min they stopped running and stepped to the sides of the treadmill belt for 15 s to enable the collection of a fingertip blood sample. The selection of test speeds was informed by the speed at LTP measured in the incremental test. Speeds used in the tests were subsequently varied by 0.5 km/h and were continued until there was sufficient data to determine MLSS, which was defined as the highest speed which resulted in an increase of blood [lactate] of less than 1 mM between 10 and 30 min (Beneke and von Duvillard 1996; Jones and Doust 1998). If the increase in blood [lactate] was ± 0.05 mM above or below 1 mM (an event that occurred in two participants), the test was repeated and the mean of the blood [lactate] at each time point during the two tests was calculated and used in the determination of MLSS.

In addition to the conventional definition of MLSS outlined above, different permutations of the criteria for the change in blood [lactate] (i.e., < 1.0 mM, < 1.5 mM and < 2.0 mM) and the time window over which changes in blood [lactate] were measured (i.e. 5–10 min, 5–15 min, 5–20 min, 5–25 min, 5–30 min, 10–15 min, 10–20 min, 10–25 min, 10–30 min, 15–20 min, 15–25 min, 15–30 min, 20–25 min and 20–30 min), were applied to provide a modified MLSS assessment.

### Determination of critical speed

The participants completed constant-speed runs to the limit of tolerance at speeds corresponding to 90%, 95%, 100% and 105% of the peak speed attained in the incremental test such that test duration was ~ 2–15 min. Pulmonary $\dot{V}{\text{O}}_{2}$ was monitored throughout the tests to assess whether end-test values exceeded 95% of the peak value determined in the step incremental test. Test durations longer than 15 min were only included in the CS determination if $\dot{V}{\text{O}}_{2}$ was greater than 95% of the respective peak value measured in the incremental test. CS was subsequently calculated from two linear regression models, the distance–time and speed–1/time models, and the non-linear hyperbolic speed–time model. The standard errors (SE) associated with the CS and Dʹ estimates for each model were calculated using regression analysis. The coefficient of variation (CoV) for each CS and Dʹ estimate was then calculated by expressing the SE as % of the parameter estimate. For a model to be accepted, the coefficient of variation (CoV) for the mathematical fit had to be < 5% for CS and < 10% for D´. The output from the model with the lowest error was used in subsequent analysis (Black et al. 2015). If the CoV was too high after the initial four tests, further tests were completed until the CoV criteria were met. In total, five participants completed four tests, two participants completed five tests and three participants completed six tests.

Following the assessment of CS and calculation of the 95% confidence intervals (CI) surrounding the CS estimate for each individual, the participants completed a final test just above CS (i.e. CS^+^ test) in which they ran to the limit of tolerance at the speed representing the upper bound of the 95% CI. The highest running speed that was used in the MLSS assessment but was below the lower bound of the 95% CI for the CS estimate for each participant was identified and defined as CS^−^.

### Statistical analysis

Analyses were performed using IBM SPSS Statistics 26.0 (Chicago, IL, USA). A two-tailed paired Students *t* test was used to analyse the difference between the running speeds at MLSS and CS. One-way repeated measures ANOVA were used to assess differences between peak values of $\dot{V}{\text{O}}_{2}$ attained during the CS determination trials and the incremental test, and also between the running speeds at conventional MLSS, modified MLSS and CS. Two-way ANOVA with repeated measures across condition and time (5, 10, 15, 20, 25 and 30 min) was used to assess differences in the blood [lactate] response for running speeds at MLSS and the speed 0.5 km/h above MLSS (termed MLSS^+^). Two-way ANOVA with repeated measures across condition and time (10 and 30 min < CS and 5 min and end-exercise > CS) was also used to assess differences in the $\dot{V}{\text{O}}_{2}$ response for running speeds at MLSS, MLSS^+^, CS^−^ and CS^+^. When sphericity was violated, the significance of F-ratios was adjusted using the Greenhouse–Geisser procedure and significant interaction and main effects were followed up using LSD post hoc tests. Linear regression analysis using Pearson product moment was carried out to determine the relationship between CS and D´ and the relationship between the running speeds at conventional MLSS, modified MLSS, and CS. Significance was set at *P* < 0.05 and results are reported as mean ± SD.

## Results

The LT and LTP occurred at 14.5 ± 1.2 and 16.8 ± 1.0 km/h, respectively, and the peak speed attained in the incremental test was 19.6 ± 1.3 km/h. The $\dot{V}{\text{O}}_{{2\,\,{\text{peak}}}}$ achieved during the incremental test was 4.65 ± 0.47 L/min or 63.0 ± 4.0 mL/kg/min.

The times to exhaustion (s) in the CS prediction trials performed at 90%, 95%, 100% and 105% of the peak speed attained in the incremental treadmill test were 801 ± 219, 500 ± 146, 388 ± 112 and 185 ± 33 s. The mean $\dot{V}{\text{O}}_{{2\,\,{\text{peak}}}}$ attained during the CS trials (4.92 ± 0.58 L/min) was not significantly different from the peak value achieved during the step incremental test (*P* = 0.28). The CS and D´ were 16.4 ± 1.3 km/h and 216 ± 79 m, respectively, and the CoV was 0.8 ± 0.7% for CS and 7 ± 3% for D´.

Following the calculation of CS and the associated 95% CI, participants completed a final test to the limit of tolerance at a speed just (~ 2.4%) above CS (i.e. at 16.8 ± 1.3 km/h; CS^+^). The time to the limit of tolerance at this speed was 17.0 ± 4.6 min and there was a strong positive correlation with D´ (r = 0.82, *P* < 0.01).

### Comparison of critical speed and running speed at maximal lactate steady state

**Fig. 1:**
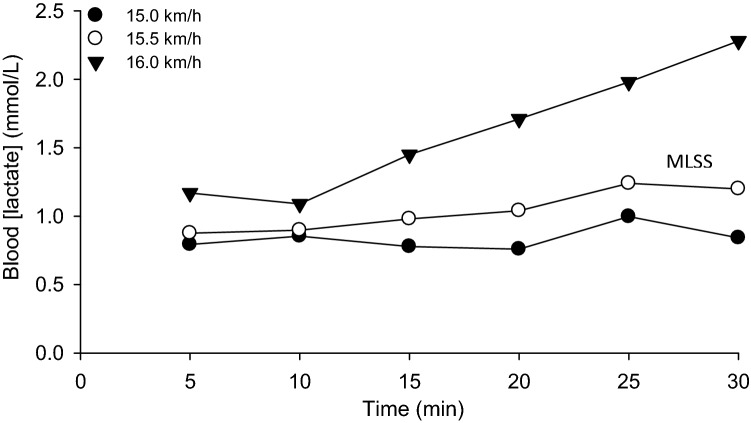
Maximal lactate steady-state (MLSS) assessment in a representative individual. MLSS was identified as the highest speed where the increase in blood [lactate] did not exceed 1 mM between 10 and 30 min

**Fig. 2:**
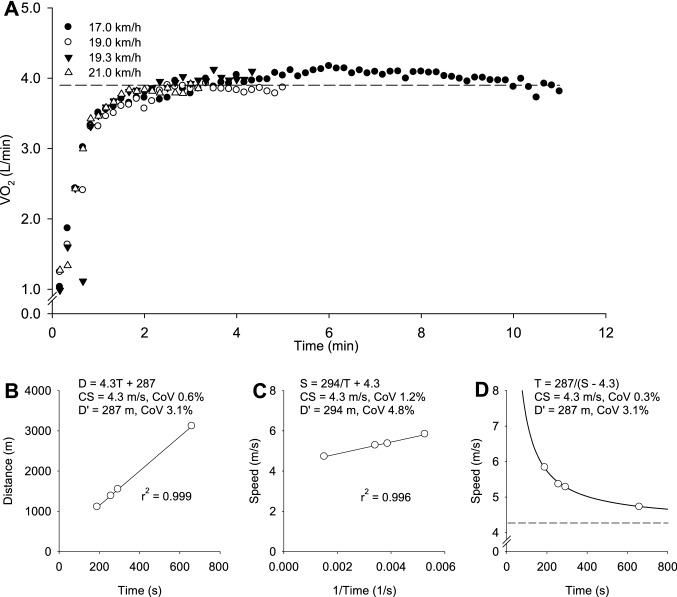
Pulmonary $\dot{V}{\text{O}}_{2}$ responses to four severe-intensity prediction trials at speeds ranging from 17.0 to 21.0 km/h (Panel **A**), and the critical speed (CS) and D´ estimation in a representative participant using distance–time (Panel **B**), speed–1/time (Panel **C**) and speed–time models (Panel **D**). The model with the lowest sum of coefficients of variation (CoV) for CS and D´ for each participant was selected for analysis. The dashed line indicates the $\dot{V}{\text{O}}_{{2\,\,{\text{peak}}}}$ measured in the initial step incremental test in panel **A**, and the speed-asymptote (CS) in panel **D**

**Fig. 3:**
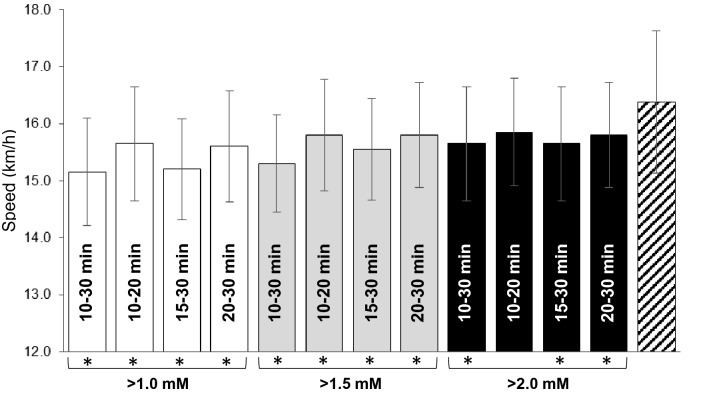
Different permutations of the maximal lactate steady state (MLSS) definition including < 1.0, < 1.5 and < 2.0 mM blood [lactate] increase, over time intervals of 10–30 min, 10–20 min, 15–30 min and 20–30 min, and the critical speed (dashed bar). Note that, for clarity, not all of the MLSS permutations are shown. All MLSS permutations were lower than CS (*P < 0.05), except for the < 2.0 mM increase in blood [lactate] between 10 and 20 min (MLSS = 15.9 ± 0.9 km/h)

The determination of MLSS is shown for a representative participant in Fig. 1 and the determination of CS is shown in Fig. 2. There was a significant difference between CS and conventionally determined MLSS (16.4 ± 1.3 vs 15.2 ± 1.0 km/h, respectively; *P* < 0.001). Different permutations of the permitted blood [lactate] increase, and the time window over which [lactate] increased, resulted in different estimates for speed at MLSS (Fig. 3). Of all of the 45 permutations of blood [lactate] increment and time window that were considered, only the criterion of a < 2.0 mM increase in blood [lactate] between 10 and 20 min produced an MLSS value (15.9 ± 0.9 km/h) that was not significantly different from CS (Fig. 3).

The conventional MLSS was significantly correlated with modified MLSS (*r* = 0.80, *P* < 0.01) and CS (*r* = 0.96, *P* < 0.001) and the modified MLSS was significantly correlated with CS (*r* = 0.80, *P* < 0.01).

### Behaviour of blood [lactate] and oxygen uptake in the proximity of MLSS and CS

**Fig. 4:**
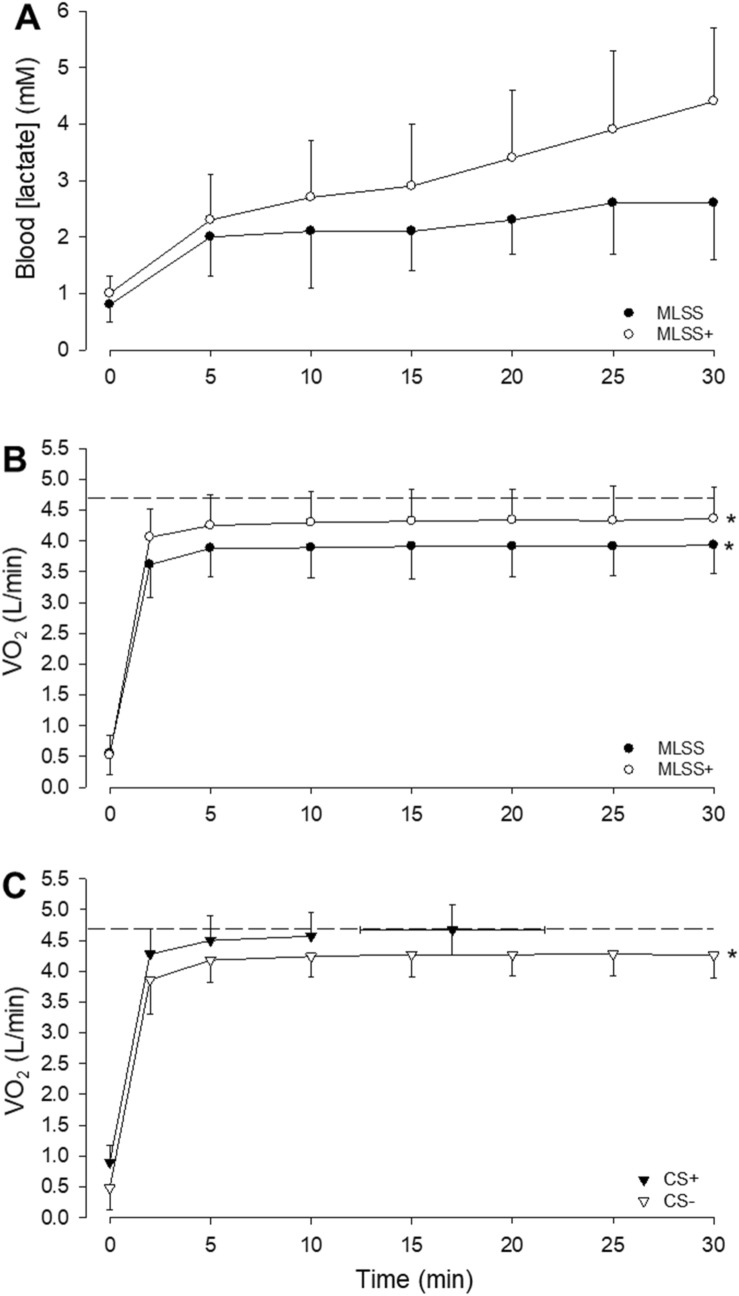
Panel **A**: blood [lactate] responses at the established maximal lactate steady state (MLSS, 15.2 ± 0.9 km/h) and at the speed immediately above MLSS (MLSS^+^; 15.7 ± 0.9 km/h). Panel **B**: pulmonary $\dot{V}{\text{O}}_{2}$ responses during exercise at MLSS and MLSS^+^; note that $\dot{V}{\text{O}}_{2}$ did not change significantly between 10 and 30 min. Panel **C**: $\dot{V}{\text{O}}_{2}$ at the speeds immediately below CS (CS^−^, 15.9 ± 0.9 km/h) and above CS (CS^+^, 16.8 ± 1.3 km/h). $\dot{V}{\text{O}}_{2}$ did not change significantly between 10 and 30 min at CS^−^ but increased between 5 min and end-exercise for CS^+^ (*P* < 0.05). The dashed line in panels B and C indicates the group mean $\dot{V}{\text{O}}_{{2\,\,{\text{peak}}}}$ measured in the step incremental test. Error bars indicate standard deviations. *End-exercise $\dot{V}{\text{O}}_{2}$ significantly different from $\dot{V}{\text{O}}_{{2\,\,{\text{peak}}}}$ measured in the step incremental test (*P* < 0.05)

There was a significant main effect by time (*F* = 13.7, *P* = 0.005) and by condition (*F* = 38.1, *P* < 0.001), and a significant interaction effect (*F* = 14.1, *P* < 0.001) on blood [lactate] across MLSS and MLSS^+^ trials. Post hoc tests showed significant differences in blood [lactate] between MLSS and MLSS^+^ trials at 10, 15, 20, 25 and 30 min (*P* < 0.05 for all; Fig. 4A). The change in blood [lactate] between 10 and 30 min was significantly greater during the run at MLSS^+^ compared to the run at MLSS (*P* < 0.001; Fig. 4A). During the MLSS run, blood [lactate] remained stable (2.1 ± 1.0, 2.3 ± 0.6 and 2.6 ± 1.0 mM at 10, 20 and 30 min, respectively) whereas, during the MLSS^+^ run, blood [lactate] increased with time (2.7 ± 1.0, 3.4 ± 1.2 and 4.4 ± 1.3 mM at 10, 20 and 30 min, respectively).

There were significant main effects by time (*F* = 15.9, *P* = 0.003) and by condition (*F* = 21.5, *P* < 0.001), and a significant interaction effect (*F* = 4.3, *P* = 0.029) on $\dot{V}{\text{O}}_{2}$ for running speeds at MLSS, MLSS^+^, CS^−^ and CS^+^. Post hoc analysis revealed that $\dot{V}{\text{O}}_{2}$ during MLSS was lower than in MLSS^+^, CS^−^ and CS^+^ (*P* = 0.001, *P* = 0.004 and *P* < 0.001, respectively), and $\dot{V}{\text{O}}_{2}$ during CS^+^ was greater than in MLSS, MLSS^+^ and CS^−^ (*P* < 0.001, *P* = 0.012 and *P* < 0.001, respectively), while there was no difference between MLSS^+^ and CS^−^ (*P* = 0.38). $\dot{V}{\text{O}}_{2}$ did not change between 10 min and end-exercise during the runs at MLSS, MLSS^+^ or CS^−^ (*P* = 0.10, *P* = 0.26, *P* = 0.78, respectively) (Fig. 4B and C). During the run at CS^+^, however, $\dot{V}{\text{O}}_{2}$ increased significantly between 5 min and the limit of tolerance (*P* < 0.001; Fig. 4C). The $\dot{V}{\text{O}}_{{2\,\,{\text{peak}}}}$ measured at the limit of tolerance in the CS^+^ run (4.67 ± 0.41 L/min) was not significantly different from $\dot{V}{\text{O}}_{{2\,\,{\text{peak}}}}$ measured in the step incremental test; however, the end-exercise $\dot{V}{\text{O}}_{2}$ in the MLSS, MLSS^+^ and CS^−^ runs were all significantly lower than $\dot{V}{\text{O}}_{{2\,\,{\text{peak}}}}$ measured in the step incremental test (*P* < 0.05; Fig. 4B and C).

## Discussion

The principal findings of the present study are that: (1) CS is higher than the speed at MLSS; (2) running at a speed 0.5 km/h above MLSS (i.e. MLSS^+^) results in a significant increase in blood [lactate] between 10 and 30 min, but no significant change in $\dot{V}{\text{O}}_{2}$ over the same time frame; (3) running at a speed ~ 0.4 km/h above CS (i.e. CS^+^), but not at a speed ~ 0.5 km/h below CS (i.e. CS^−^), results in a significant increase in $\dot{V}{\text{O}}_{2}$ over time with peak $\dot{V}{\text{O}}_{2}$ attained at the limit of tolerance; and (4) in the current data set, defining the MLSS as a < 2 mM increment in blood [lactate] between 10 and 20 min, as opposed to a < 1 mM increment between 10 and 30 min as per the conventional definition, eliminates the difference between MLSS and CS. These findings are consistent with our experimental hypotheses. We interpret the results to indicate that, while MLSS differentiates running speeds for which the blood [lactate] response is steady state vs. non-steady state, it underestimates the maximal metabolic steady state as represented by the ability, or inability, to stabilise $\dot{V}{\text{O}}_{2}$ during exercise. In contrast, running at a speed just above, but not just below, CS results in a rising $\dot{V}{\text{O}}_{2}$ profile until the limit of tolerance is reached, indicating that CS provides a more appropriate representation of maximal metabolic steady state.

Consistent with our hypothesis, CS was significantly higher than the speed at MLSS. This finding is in agreement with several other studies which have found CS or CP to be higher than MLSS, both for running and cycling (Dekerle et al. 2003, 2005; Mattioni Maturana et al. 2016; Pringle and Jones 2002). In the present study, CS was ~ 8% higher than MLSS, which is broadly consistent with previous comparisons: for example, 4% in Smith and Jones (2001), 9% in Pringle and Jones (2002), 16% in Dekerke et al. (2003), 5% in Dekerke et al. (2005) and 1% in Keir et al (2015). While differences in experimental protocol including the number and duration of prediction trials, the sensitivity of MLSS determination (which is a function of the power or speed increments between trials), and the mathematical models used to calculate CP or CS may explain some of the discrepancy (Bishop et al. 1998; Black et al. 2015; Mattioni Maturana et al. 2018), it is now clear that while CP and MLSS have historically been considered to represent broadly the same phenomenon, there is limited agreement in practice.

The tendency for CP to be higher than MLSS has led to the interpretation that CP overestimates the maximal metabolic steady state, with the assumption that MLSS represents the ‘gold standard’ and that a blood [lactate] steady state reflects a $\dot{V}{\text{O}}_{2}$ steady state (e.g. Iannetta et al. 2018; Pringle and Jones 2002). However, the physiological rationale for the accepted definition of MLSS (i.e. the highest power or speed at which blood [lactate] does not increase by more than 1 mM between 10 and 30 min of exercise, equivalent to a 0.05 mM increment in blood [lactate] per min) is obscure and apparently arbitrary (Jones et al. 2019a). It has been pointed out that reliance on blood [lactate] alone as a proxy for the existence of muscle metabolic and systemic homeostasis is hazardous; that absolute blood [lactate] is influenced by exercise-induced haemoconcentration and modifications to substrate metabolism (Tanaka 1991); and that human, technical and instrument error in the collection and analysis of capillary blood samples for [lactate] (Morton et al. 2012; Tanner et al. 2010) at just two discrete time points (10 and 30 min), along with poor day-to-day reproducibility of [lactate] during MLSS assessment (Hauser et al. 2013), could result in either false positives or false negatives (Jones et al. 2019a). Moreover, the use of discrete powers or speeds in the MLSS assessment procedure will inevitably result in an underestimation of the actual maximal metabolic steady state (Jones et al. 2019a), the extent of which will depend on the sensitivity of the measurements, with tests typically differing by 20–30 W for cycling and 0.5 or 1.0 km/h for running.

Methodological strengths of the present study included that: at least three (and frequently 4 or 5) 30-min trials were used in the assessment of MLSS; the trials were separated by relatively small (0.5 km/h) running speed increments; and, where the increase in blood [lactate] was within 0.05 mM of meeting the criterion of a 1 mM increase (an event that occurred in two participants), the test was repeated and the mean response was used in subsequent analysis. These elements of the study design provide a high level of confidence in the precision of MLSS determination. An important finding in the present study was that, while MLSS partitioned a running speed at which blood [lactate] did not change between 10 and 30 min from a running speed at which blood [lactate] increased significantly over the same time frame, it did not separate steady state from non-steady-state $\dot{V}{\text{O}}_{2}$ responses. Specifically, at the speed immediately (0.5 km/h) above that which was identified as representing MLSS, $\dot{V}{\text{O}}_{2}$ did not change significantly between 10 and 30 min. Other recent studies also indicate that MLSS does not reflect the maximum power or speed at which $\dot{V}{\text{O}}_{2}$ can be stabilised. For example, Bräuer and Smekal (2020) measured MLSS from 4 to 6 30-min cycle exercise tests in 45 participants and reported stable $\dot{V}{\text{O}}_{2}$ over the last 10 min of exercise at both MLSS and at a power output above MLSS. Similarly, Iannetta et al (2018) found that when participants cycled at 10 W above the power output established as representing MLSS, a $\dot{V}{\text{O}}_{2}$ steady state was manifest. Such results are insightful because it is known that, in the steady state, pulmonary $\dot{V}{\text{O}}_{2}$ closely reflects skeletal muscle $\dot{V}{\text{O}}_{2}$ (Grassi et al. 1996; Krustrup et al. 2009). Moreover, pulmonary $\dot{V}{\text{O}}_{2}$ and intramuscular [PCr] profiles are closely related both when steady states can be attained and when slow components in the responses are manifest (Rossiter et al. 2002). Collectively, these findings indicate that MLSS, as conventionally defined, does not represent the maximal metabolic steady state, which is more appropriately defined in relation to the ability to stabilise pulmonary $\dot{V}{\text{O}}_{2}$ and thus skeletal muscle $\dot{V}{\text{O}}_{2}$ and [PCr] (Grassi et al. 1996; Rossiter et al. 2002).

In contrast, when the athletes in the present study ran at a speed just above CS (CS^+^, 16.8 ± 1.3 km/h, calculated according to the 95% CI surrounding the estimate of CS), $\dot{V}{\text{O}}_{2}$ increased significantly beyond 5 min, and the end-exercise $\dot{V}{\text{O}}_{2}$ was not different from the $\dot{V}{\text{O}}_{{2\,\,{\text{peak}}}}$ measured in the maximal incremental test. When the highest speed below CS the athletes ran at (CS^−^, 15.9 ± 0.9 km/h) was considered, $\dot{V}{\text{O}}_{2}$ was not significantly different between 10 and 30 min. Therefore, when the athletes ran at a speed that was ~ 6 s per km slower than CS, $\dot{V}{\text{O}}_{2}$ was in steady state and the prescribed 30 min of exercise was completed, whereas when the athletes ran at a speed that was ~ 5 s per km faster than CS, a $\dot{V}{\text{O}}_{2}$ steady state could not be achieved and exercise tolerance was limited to ~ 17 min, indicative of exercise within the severe-intensity domain (Black et al. 2017; Poole et al. 1988).

These data indicate that CS provides a rather precise demarcation of the highest running speed at which $\dot{V}{\text{O}}_{2}$ can be stabilised. This observation is consistent with several previous studies which indicate that CP or CS is the metabolic threshold which partitions severe-intensity exercise, which, by definition, is characterised by an inexorable increase in $\dot{V}{\text{O}}_{2}$ to its peak value at the limit of tolerance, from heavy-intensity exercise, during which a $\dot{V}{\text{O}}_{2}$ steady state can still be achieved (Jones et al. 2010; Poole et al. 2016; Whipp 1994). It has been established from muscle biopsy studies that these characteristic $\dot{V}{\text{O}}_{2}$ profiles during exercise performed above and below CP are associated with corresponding steady-state or non-steady-state responses in skeletal muscle PCr and lactate concentrations (Black et al. 2017; Vanhatalo et al. 2016). These findings are reinforced by non-invasive ^31^P-magnetic resonance spectroscopy assessment of the skeletal muscle metabolic responses, which demonstrate striking differences in the profiles of PCr, inorganic phosphate and pH for exercise performed just above, compared to just below, CP (Jones et al. 2008). These differences in the rates of substrate utilisation and metabolite accumulation likely underpin observations that the rate and nature of neuromuscular fatigue development also differ according to the intensity of the exercise task relative to CP (Black et al. 2017; Burnley et al. 2012; Dinyer et al. 2020; Pethick et al. 2020). Finally, it is pertinent to note that simultaneous assessment of the responses of muscle [lactate] and blood [lactate] during heavy-intensity and severe-intensity exercise reveal that the former may be stable while the latter rises (Jones et al. 2019b), suggesting differences in the dynamics of lactate accumulation in the muscle and blood compartments. These observations clearly indicate that a maximum blood [lactate] steady state will likely underestimate the maximal metabolic steady state as determined by muscle [lactate], as well as the responses of other muscle ions and metabolites, and $\dot{V}{\text{O}}_{2}$.

The evaluation of CP or CS is not without its challenges, ideally requiring 3–5 maximal efforts on separate days, although this burden can be alleviated in athletes through the use of recent training or competition data (Jones and Vanhatalo 2017; Karsten et al. 2015; Smyth and Muniz-Pumares 2020). In some situations, estimating CP or CS from submaximal exercise tests may be considered preferable to direct assessment. While it is clear from both the present study and from earlier studies that the conventional protocol and criteria for MLSS assessment underestimates the maximal metabolic steady state, it is possible that adjustments to these factors might enable a closer approximation of CP or CS. In the present study, we calculated the running speed at MLSS using a variety of permutations of absolute increments in blood [lactate] (e.g. 1.0, 1.5 and 2.0 mM) and the time frame over which such increments were considered. Of these permutations, we found that modifying the criteria to a 2 mM increment in blood [lactate] between 10 and 20 min increased the group mean running speed at MLSS from 15.2 to 15.9 km/h and eliminated the difference between MLSS and CS. This approach does not, however, circumvent other limitations to relying solely on measurements of blood [lactate] for the assessment of the maximal metabolic steady state and might be considered to be just as obscure and arbitrary as the conventional definition of MLSS. At the present time, therefore, we favour direct assessment of CP or CS if precision is required in scientific studies or for training prescription.

By definition, exercise in the severe-intensity domain should result in the attainment of $\dot{V}{\text{O}}_{{2\,\,{\text{peak}}}}$ at or shortly before the limit of tolerance is reached (Hill et al. 2002; Jones et al. 2010; Poole et al. 1988; Whipp 1994). It should be appreciated, however, that CP and CS are estimated mathematically from several prediction trials and there will inevitably be some error, both computational and biological (e.g. day-to-day variability), surrounding the estimates (Black et al. 2015; Mattioni Maturana et al. 2018). For this reason, asking participants to exercise to the limit of tolerance exactly at the computed CP or CS can result in wide variability in both the physiological responses and the time to the limit of tolerance due to some participants being below and others being above the CP or CS (Pethick et al. 2020; see Jones et al. 2019a for review). Indeed, the notion of exercising at the CP (or CS) is vacuous because the asymptote of the power–time relationship represents the power that lies exactly between those powers at which W´ is utilised and those powers at which it is not; that is, it defines the threshold separating the heavy-intensity and severe-intensity domains and the inherent steady-state or non-steady-state physiological behaviour that defines those domains; and therefore, it is erroneous to define CP as the highest power at which steady-state responses are observed. In the present study, we employed several approaches to minimise the error surrounding the CS estimate including: having the athletes complete at least 4 and up to 6 prediction trials; ensuring that the athletes ran to the limit of tolerance during all prediction trials, as validated by there being no significant difference in $\dot{V}{\text{O}}_{{2\,\,{\text{peak}}}}$ achieved in the prediction trials compared to the maximal incremental test; and applying all three standard mathematical models and choosing the output from the model with the least error for each individual (Black et al. 2015). Together, these approaches resulted in CoV that were appreciably lower for both CS (0.8 ± 0.7%) and D´ (7 ± 3%) than the degree of error which has been suggested to be acceptable (< 5% for CS and < 10% for D´; Hill 1993).

The experimental procedures employed in the present study also ensured that the 95% CI surrounding CS were relatively narrow (i.e. group mean of ± 0.4 km/h). Our study participants, therefore, ran very close to, but very slightly above, their CS to the limit of tolerance as a validation that CS represents the heavy-to-severe exercise intensity boundary. It should be acknowledged that, while the group mean CS was estimated to be 16.4 km/h, when the 95% CI is taken into account, the ‘actual’ CS could have occurred anywhere between 16.0 and 16.7 km/h. We took appropriate measures to minimise errors arising from biological factors and mathematical modelling, but it is important to note that it is not possible to entirely eliminate the error margin surrounding any physiological threshold estimate (Pethick et al. 2020). It should also be appreciated that this range of speeds within which the CS resides represents an error of only ± 4–5 s per km (~ 2%).

The time to the limit of tolerance at CS^+^ (17.0 ± 4.6 min) was closely correlated with the athletes’ D´ (*r* = 0.82). This indicates that when an exercise task is relativized to athletes’ CS values, then the energetic reserve or work capacity above CS becomes an important factor determining exercise tolerance. Within the limitations of the present study (i.e. the error margin surrounding the estimation of CS), these results also reveal that the longest an athlete can run at a constant speed and still attain $\dot{V}{\text{O}}_{{2\,\,{\text{peak}}}}$ is approximately 17 min. Therefore, assuming an even pace is employed throughout the race to minimise the time taken to complete the distance (Fukuba and Whipp, 1999), it appears likely that athletes will attain $\dot{V}{\text{O}}_{{2\,\,{\text{peak}}}}$ during a 5000 m race (since this will be at the lower end of the severe-intensity domain) but not during a 10,000 m race (which is positioned at the upper end of the heavy-intensity domain). These results highlight an important conceptual issue: it is inappropriate to consider peri-CS (or CP) exercise to be ‘fatigueless’, or to be sustainable indefinitely or for some arbitrary time period such as 60 min; indeed, this might be considered a misinterpretation of the original descriptions of the concept (Monod and Scherrer 1965). Rather, contemporary understanding is (or, at least, in our view, should be) that CS separates exercise domains within which: (1) physiological (including muscle metabolic and cardiorespiratory) responses are differentiated by steady-state vs. non-steady-state behaviour; and (2) the predominant determinants of fatigue are altered, with exercise tolerance > CS being predictable as a function of CS and D´.

In conclusion, this study affirms that CS occurs at a higher speed than MLSS in well-trained runners. An important novel finding was that a $\dot{V}{\text{O}}_{2}$ steady state was elicited when the athletes ran at a speed which was above MLSS but below CS, whereas a $\dot{V}{\text{O}}_{2}$ steady state could not be attained when they ran at a speed which was just above CS. These results indicate that CS, rather than MLSS, provides a better representation of the maximal metabolic steady state.
